# Incarcerated Bladder Diverticulum in a Femoral Hernia Presenting as Recurrent Hematuria

**DOI:** 10.7759/cureus.9681

**Published:** 2020-08-12

**Authors:** Saptarshi Biswas, Emma M Morel, Kirklen Petersen, Austin McCrae

**Affiliations:** 1 Trauma and Acute Care Surgery, Grand Strand Medical Center, Myrtle Beach, USA; 2 General Surgery, Edward Via College of Osteopathic Medicine-Carolinas, Myrtle Beach, USA; 3 Trauma, Grand Strand Medical Center, Myrtle Beach, USA; 4 General Surgery, Grand Strand Medical Center, Myrtle Beach, USA

**Keywords:** femoral hernia, bladder diverticulum, incarcerated bladder, hematuria

## Abstract

Groin hernias are extremely common surgical pathologies and usually contain intra-abdominal viscera surrounded by peritoneum. Femoral hernias are the least common types of hernia and are predominately found in females. In rare cases, an extraperitoneal organ may be pulled into the hernia sac to become part of the content. Urinary bladder diverticulum should be considered as a possible femoral hernia content in elderly patients presenting with recurrent symptoms of lower urinary tract infections and hematuria. A high index of suspicion followed by appropriate imaging assists in making a correct preoperative diagnosis and improves postoperative outcomes. We present an uncommon case of herniation of a urinary bladder diverticulum into a femoral hernia presenting with recurrent hematuria in an elderly female.

## Introduction

Groin hernia is a common surgical pathology usually containing intra-abdominal viscera surrounded by the peritoneum. An extraperitoneal organ is not typically found within the hernia sac. However, in certain rare cases, like our case of a bladder diverticulum, extraperitoneal organs can be pulled by the sac itself and become a component of the hernia [1].

Femoral hernias, although more predominant in females, are less common than inguinal hernias and are usually complicated with incarceration or strangulation of the organ that they contain [2,3]. Bladder diverticula form within a trabeculated high-pressure urinary bladder caused by bladder outlet obstruction. In the majority of cases, bladder outlet obstruction is a disease of the male patient caused by benign prostatic hypertrophy. The clinical features of the bladder diverticulum are non-specific; hence, a high index of suspicion along with proper imaging studies is of utmost significance in making a timely diagnosis.

We present an uncommon case of a urinary bladder diverticulum that herniated into the right femoral canal which presented with recurrent bouts of hematuria.

## Case presentation

A 65-year-old female patient presented to the emergency department (ED) complaining of pelvic pressure and hematuria. Although the history was somewhat limited due to dementia, the patient did report discomfort in the suprapubic area. She denied vomiting, chest pain, shortness of breath, fevers, or chills. 

Past medical history comprised atrial fibrillation, hypertension, and stroke. Past surgical history included left cataract extraction and closed reduction of right humerus fracture. She was anticoagulated with apixaban. 

The patient was oriented in time, place, and person and appeared in no marked distress. The heart was in regular rate and rhythm. The lungs were clear with equal breath sounds bilaterally. The abdomen was soft, and there was some slight suprapubic tenderness but no masses, rebound, or guarding. Extremities were atraumatic. A 12-systems review was essentially negative except for genitourinary which was positive for hematuria. The urine specimen was cloudy in appearance with marked leukocyte esterase, 3+ protein, 2+ blood, 3+ red blood cells (RBCs), white blood cells (WBCs), too numerous to count bacteria, many squamous epithelial/low-power field (LPF), and moderate transitional epithelial cells. 

**Figure 1:**
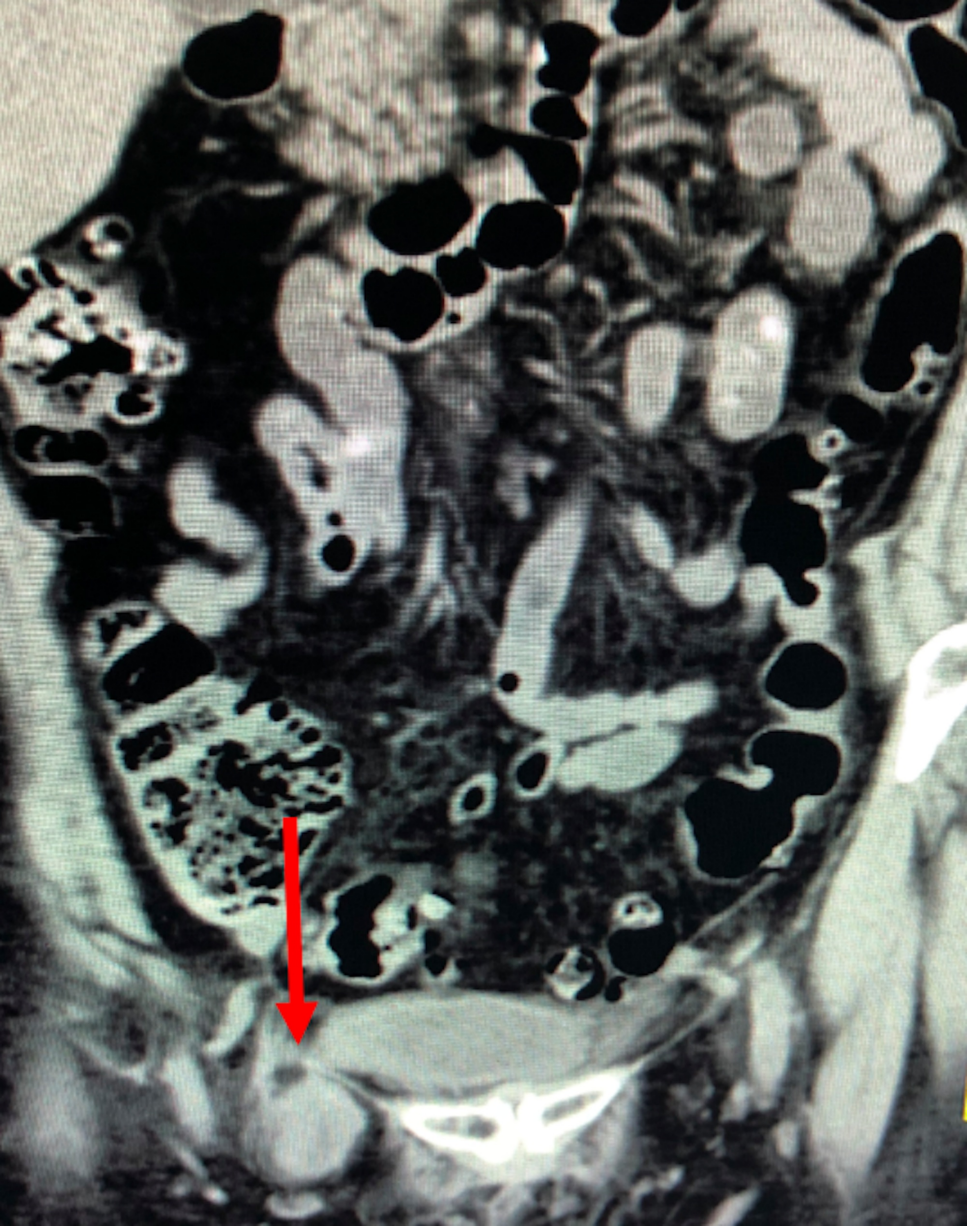
CT abdomen/pelvis coronal view: the arrow indicating herniated bladder

A CT scan of the chest, abdomen, and pelvis was completed (Figure 1).

**Figure 2:**
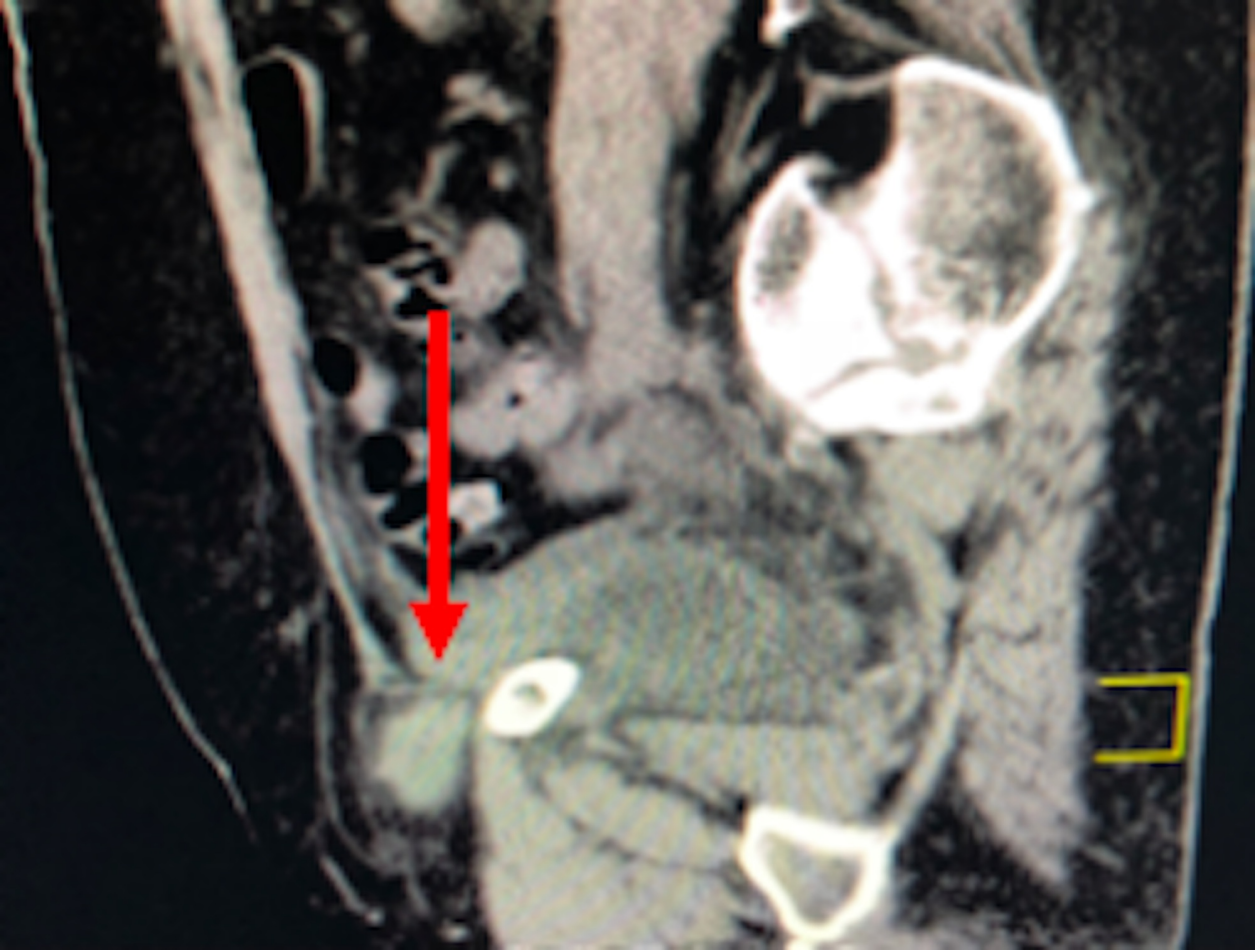
CT abdomen/pelvis sagittal view: the arrow indicating herniated bladder diverticulum

The kidneys were normal in size, configuration, and location. There was a right “urinary bladder ear” likely relating to a femoral or less likely inguinal herniation. The involved segment exhibited gross irregular mural thickening with adjacent infiltration (Figure 2).

The entrapped and adjacent mural segment was significantly thickened with adjacent infiltration with a high suspicion for strangulation/infarction. The remainder of the urinary bladder exhibited a lesser degree of mural thickening. Bilateral non-obstructing renal calculi with mild bilateral pelvocaliectasis and a Bosniak type 2 right renal cyst were also noted. The gallbladder, biliary tree, liver, spleen, pancreas, and adrenal glands were free of abnormalities as were the gut, mesenteric, nodal, and bony structures. No intraperitoneal mass or fluid or other pelvic bony or soft tissue abnormalities were noted. There was normal heart size with mitral annulus calcification. Osteopenia and thoracolumbar spondylosis were particularly advanced at L2-3 and L5-S1.

**Figure 3:**
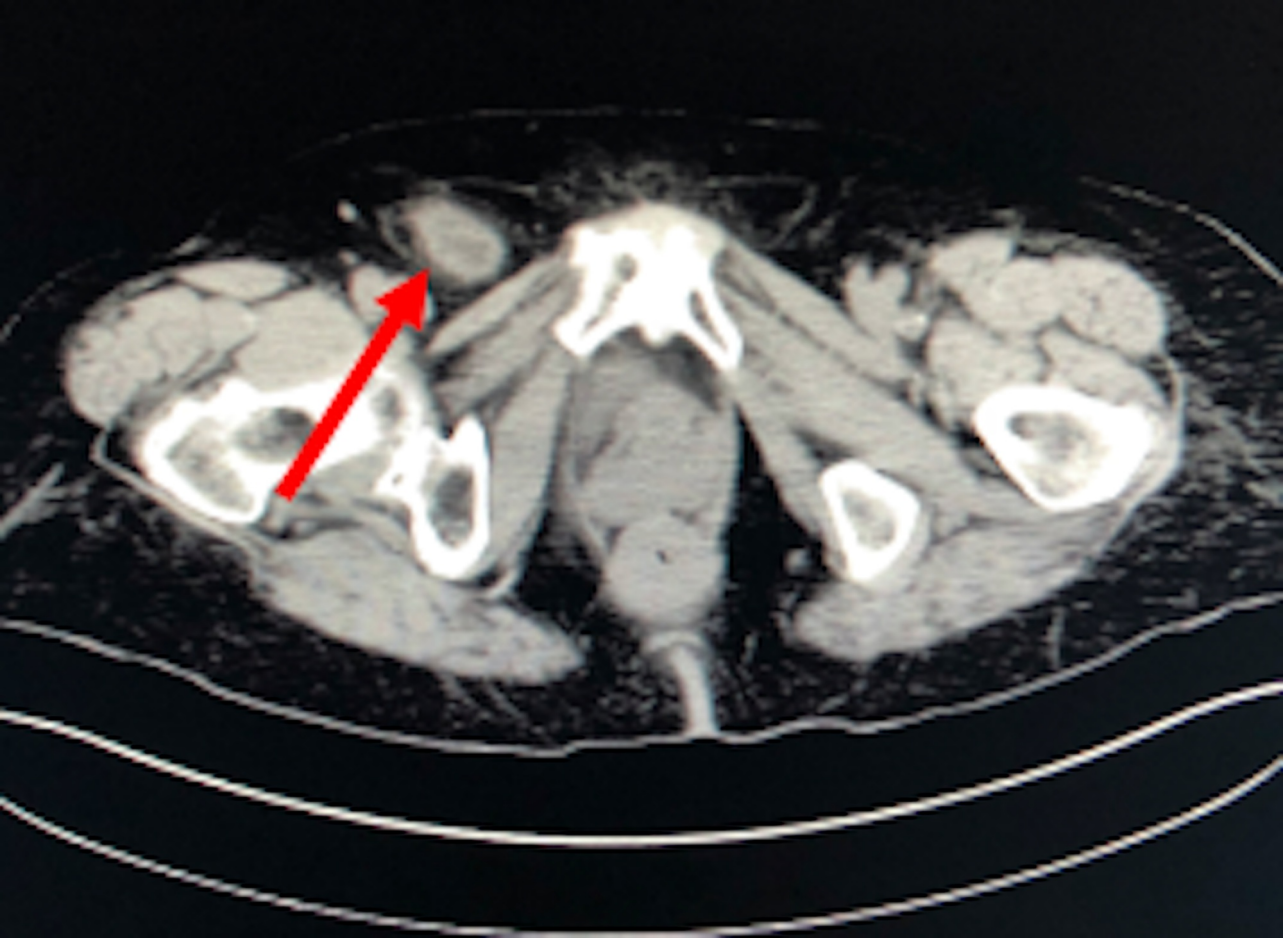
CT abdomen/pelvis axial view: the arrow indicating herniated bladder diverticulum

Prior to transport to the operating room (OR), the patient had received IV rocephin in the ED. In the OR, she was induced with general anesthesia. The skin and deeper tissue halfway between the anterior superior iliac spine and pubic tubercle were infiltrated with local anesthetic. A 5-cm oblique incision was performed parallel to the inguinal ligament and extended down through subcutaneous tissue with cautery to the level of the hernia sac which was dissected circumferentially and freed. It led to the femoral hernia defect medial to the femoral vessels (Figure 3).

It measured 1.2 cm in diameter. The herniated tissue consisted of a diverticulum of the bladder with surrounding fatty tissue. It was soft and viable and was gradually pushed back into the pelvis. The preperitoneal space was circumferentially cleared with a sponge. The repair was done by introducing a large mesh plug through the defect into the preperitoneal space. It was attached to the inguinal ligament superiorly and to the medial tissue with a couple of interrupted 2-0 Polysorb sutures. Scarpa's fascia was closed with several interrupted 3-0 Polysorb sutures. The incision was closed with a continuous subcuticular 4-0 Polysorb suture. The area was circumferentially infiltrated with 10 cc of 0.5% plain Marcaine. A sterile dressing was taped in place. 

The patient tolerated the procedure well. The patient did well postoperatively, tolerating a regular diet, and her pain was adequately controlled with an oral regimen. She was discharged home on postoperative day 1. The patient was seen one week postop in the outpatient setting where she was doing well. She returned for a one-month postop appointment where she continued to be without complaints, and she has since been lost to follow-up. 

## Discussion

Bladder diverticula are relatively rare and are usually caused by weakening of muscular fibers of the bladder and increased intravesicular pressure leading to herniation of the bladder mucosa through the bladder wall [4]. It is typically seen in males where the infravesical obstruction is caused by benign prostatic hypertrophy. In the setting of a groin hernia, a long-standing history of difficult urination, incomplete voiding, and/or straining, as seen in these elderly males, warrants suspicion for the diagnosis of a sliding inguinoscrotal hernia containing the urinary bladder or a bladder diverticulum. We report a case of a herniated bladder diverticulum within a femoral hernia in a female patient, which is truly rare. Review of current literature revealed only two cases reported prior to this one, both of which were male patients [4].

Bladder diverticulum can be congenital, acquired, or iatrogenic. Congenital cases are almost exclusively in boys, usually singular and large, and sometimes associated with congenital neurodegenerative disorders, such as Ehlers-Danlos syndrome and Menkes’ syndrome [5]. Acquired bladder diverticula are usually connected to outlet obstruction. Prostatic hyperplasia, posterior urethral valves, urethral stricture, neuropathic bladder dysfunction, and dyssynergia of the urethral sphincter are some of the possible etiologies of the bladder outlet obstruction [6]. In young women with functional bladder outlet obstruction, important causes are spastic urethral sphincter and impaired relaxation of pelvic floor musculature during voiding, which result in difficulty voiding, urinary retention, and overactive bladder [7]. Iatrogenic bladder diverticula are commonly caused by catheter insertion. Medications are the mainstay of treatment, along with biofeedback, pelvic floor muscle training, and botulinum toxin A injection. Surgical treatment may be necessary in recurrent urinary tract infections, bladder calculi, urethral obstruction, or malignancy [8]. Intermittent self-catheterization in urinary retention or augmentation cystoscopy in cases of decreased bladder capacity may be warranted [6].

Groin hernias are usually diagnosed clinically. However, it is imperative to know the true location, its relationships, and content characteristics prior to surgical intervention [4]. Although some authors have used ultrasonography to determine the hernia contents because of its non-invasive properties, CT scan is widely regarded as the diagnostic modality of choice [9,10]. In addition to localization, CT scan provides valuable information regarding both the characteristics of the contents and the relationship to the inferior epigastric vessels [11,12]. Some authors advocate for use of cystogram during the preoperative evaluation to assess the urinary bladder anatomy and the degree of involvement [13]. 

Either an extraperitoneal or an intraperitoneal approach may be employed for the surgical management of a bladder diverticulum herniated through the femoral or inguinal canals [1]. We opted for the open surgical method. Some authors have mentioned the open technique as the preferred method although there are reported cases of laparoscopic repair [14,15]. The surgeon’s preference, expertise with advanced laparoscopy, local status, and existent comorbidities of the patient play a role in approach determination. We prefer to use a mesh repair. Preoperative Foley catheterization is advisable. Proper identification of the hernia and individual relationship of each content is important [15]. In male patients where bladder diverticuli are due to benign prostatic hypertrophy, diverticulectomy and simple prostatectomy can be performed simultaneously. Depending on availability, a urology consultation may be required. If the bladder has herniated, it is reduced and repositioned in its original anatomical position [16]. Bladder resection may be indicated in the case of hernioplasty, bladder neck necrosis, bladder tumors, bladder diverticulum, and hernia neck less than 5 cm. Some data suggest the bladder may be damaged in approximately 12% of inguinal bladder hernias, which decreases if proper preoperative diagnosis is done with appropriate imaging.

## Conclusions

Although rare in females, urinary bladder diverticulum should be considered as a possible femoral hernia content in elderly patients presenting with recurrent symptoms of lower urinary tract infections and hematuria. A high index of suspicion along with appropriate imaging assists in making the correct preoperative diagnosis and improves postoperative outcomes. Intraoperatively, the bladder should be checked for iatrogenic injuries and repaired. Inguinal bladder hernia is sometimes associated with urological malignancies which, if suspected, warrant appropriate follow-up. 
